# Distinctive Behaviors of Druggable Proteins in Cellular Networks

**DOI:** 10.1371/journal.pcbi.1004597

**Published:** 2015-12-23

**Authors:** Costas Mitsopoulos, Amanda C. Schierz, Paul Workman, Bissan Al-Lazikani

**Affiliations:** Cancer Research UK Cancer Therapeutics Unit, The Institute of Cancer Research, London, United Kingdom; Boston University, UNITED STATES

## Abstract

The interaction environment of a protein in a cellular network is important in defining the role that the protein plays in the system as a whole, and thus its potential suitability as a drug target. Despite the importance of the network environment, it is neglected during target selection for drug discovery. Here, we present the first systematic, comprehensive computational analysis of topological, community and graphical network parameters of the human interactome and identify discriminatory network patterns that strongly distinguish drug targets from the interactome as a whole. Importantly, we identify striking differences in the network behavior of targets of cancer drugs versus targets from other therapeutic areas and explore how they may relate to successful drug combinations to overcome acquired resistance to cancer drugs. We develop, computationally validate and provide the first public domain predictive algorithm for identifying druggable neighborhoods based on network parameters. We also make available full predictions for 13,345 proteins to aid target selection for drug discovery. All target predictions are available through canSAR.icr.ac.uk. Underlying data and tools are available at https://cansar.icr.ac.uk/cansar/publications/druggable_network_neighbourhoods/.

## Author Summary

The need for well-validated targets for drug discovery is more pressing than ever, especially in cancer in view of resistance to current therapeutics coupled with late stage drug failures. Target prioritization and selection methodologies have typically not taken the protein interaction environment into account. Here we analyze a large representation of the human interactome comprising almost 90,000 interactions between 13,345 proteins. We assess these interactions using an extensive set of topological, graphical and community parameters, and we identify behaviors that distinguish the protein interaction environments of drug targets from the general interactome. Moreover, we identify clear distinctions between the network environment of cancer-drug targets and targets from other therapeutics areas. We use these distinguishing properties to build a predictive methodology to prioritize potential drug targets based on network parameters alone and we validate our predictive models using current FDA-approved drug targets. Our models provide an objective, interactome-based target prioritization methodology to complement existing structure-based and ligand-based prioritization methods. We provide our interactome-based predictions alongside other druggability predictors within the public canSAR resource (cansar.icr.ac.uk).

## Introduction

Identifying novel drug targets and prioritizing proteins for target validation and therapeutic development are essential activities in modern mechanism-driven drug discovery, and are key if we are to benefit from large-scale genomic initiatives [1]. Multiple approaches exist to estimate the ‘druggability’ or chemical tractability of a protein [2–4]. 3D structure-based assessments predict cavities in the protein structure that are capable of binding small molecules [3–5]. Alternative methods include sequence feature-based druggability [4,6] and ligand-based methods that examine the properties of compounds known to be bioactive against a protein [7–9].

While many genes have been identified as disease-causing (see for example reports on cancer [10,11] and cardiovascular disease [12]), the products of relatively few of these have become targets for approved therapeutics. The challenges facing researchers attempting to target a gene and its product proteins for clinical application lie both in validating their pathogenic role and in their technical ‘doability’. As well as possessing a pocket or interface suitable for drug binding, a potential drug target must exert an appropriate influence on the system, enabling a drug to have a selective and enduring therapeutic effect. Genetic diseases, prominently cancer, are disorders arising from deregulation or disruption of normal cellular wiring and protein communication. It is therefore essential that the network environment of a potential drug target should be incorporated into target selection rationale.

Previous studies have highlighted the importance of considering the interactome when predicting protein function [13,14], assessing drug-target interaction data and understanding polypharmacology [9,15], or predicting novel uses for drugs [16–18]. Meanwhile, recent technological advances in systems biology have generated large quantities of experimentally-derived protein interaction data [19] and networks have been applied to understand the relationships between these protein interactions and disease [20–24]. For example, relationships between protein interactions and cancer have been identified by integrating protein interaction networks with functional or gene expression data [25,26]; structural differences in the network between cancer-causing and non-cancer-causing genes have been highlighted [24–26]; and a potential core ‘diseasome’ network has been documented [27].

Tantalizingly, a number of studies have examined the distribution of some focused topological network parameters, such as degree and clustering coefficient, in drug targets versus non-drug targets [17,18,28]. Most notably, the number of first neighbors (degree) was identified as a distinguishing feature of the human ‘highly optimized tolerance’ or ‘HOT’ network [17] and was proposed as a measure to consider when selecting drug targets. This proposition was based on the assumption that inhibiting proteins with a high degree will impact widely on a biological system and thus have undesirable effects [17]. While such extrapolations may not always hold true—for example, many cancer-drug targets are major hubs yet their modulation, singularly or in combination, shows clear selectivity for cancer cells (see references [29,30] and discussed below)—these studies have highlighted the potential of network parameters to provide discriminatory patterns for identifying druggable network nodes. However, such studies imply that any patterns that may exist to distinguish drug targets from other proteins are likely to be complex. Indeed, no purely network-based discriminatory models have been described. Instead, reported models include functional or family annotation [4,6,17,18,28,31] to ensure predictive power. These functional and family annotations overshadow any network parameters due to the dominance of certain protein families (e.g. G-protein coupled receptors) or functions (e.g. enzymes) in the training set of known drug targets [7–9,32].

Thus, the true network behaviors that may distinguish the points in a network most suitable for therapeutic intervention remain elusive. Consequently, despite the fundamental role of cellular wiring in drug action and resistance, there is, to our knowledge, no network-based druggability predictor in existence in the public domain. In this article, we present a comprehensive and systematic computational analysis of 321 topological, community-based and graphical network properties of a fully-connected human interactome. Furthermore, we show how these properties relate to druggability in its more complete sense: the suitability for intervention with a molecularly targeted therapeutic agent of any type. In particular, we explore the differing network environments of cancer-drug targets and targets from other therapeutic indications and discuss the potential impact that these differing network environments may have on resistance to cancer drugs. We build and benchmark the first publicly available predictive network-based models to identify likely druggable network nodes and node clusters, and apply these models to a set of 13,345 proteins in the human interactome. To our knowledge, these are the first published models that enable a prediction of druggability based on the topological, graphical and community behavior of proteins in the interaction network. Our network method is intended to be used to complement structure-, sequence- and ligand-based druggability prediction methods in order to provide a holistic view of the likely utility of a given protein as a drug target. The results of this analysis have been implemented in the canSAR knowledgebase [10,11,33,34] and each protein’s network signature, as well as its predicted druggability score, is accessible alongside other druggability measures at http://cansar.icr.ac.uk/.

## Results

Having constructed the high quality interactome, we constructed four distinct, manually curated training sets representing: a) all targets of FDA-approved drugs, which in turn was divided into b) targets of cancer drugs and c) targets of drugs from non-cancer therapeutic areas; and finally d) cancer proteins—the products of cancer associated genes. We then trained suites of predictive models using each of these training sets to predict druggability using only network topological, community and graph features (see Methods and Fig A in S1 Text).

Our predictive models achieve a mean area-under-the-curve (AUC) value of 83% (see also Fig E in S1 Text for recall precision). The significance compared to random prediction is as follows: drug targets (all therapeutics areas), p-value < 2.0^−16^ compared to 0.018 for the randomized network model; cancer drug targets, p-value = < 2.0^−16^ compared to 0.125 for the randomized network model and non-cancer-drug targets, p-value = < 2.0^−16^ compared to 0.331 compared to the randomized network model. Thus, the models have high predictive power using extensive *in silico* validation and can, therefore, be used to enrich potential drug targets during target prioritization for drug discovery. The full results are provided in S1 Table and per-protein analyses are supplied within the canSAR resource (https://cansar.icr.ac.uk/) alongside previously described structure-based and ligand-based methodologies [4,12,34].

### Network topology differentiates cancer-drug targets

We found that several network parameters show distinct distributions in drug targets or targets of cancer versus non-cancer drugs (key parameters are shown in Fig C in S1 Text). On average, drug targets have a higher degree (i.e. more first neighbors) than non-drug targets. Whilst the mean degree of drug targets is 26.34, this is primarily due to cancer targets which have a mean degree of 47.21 (compared to 13.72 for targets from other therapeutic areas (TAs) and 12.65 for the background—see Table C in S1 Text). We also found that targets of cancer therapeutics have more neighbors and tend to be more hub-like than the average cancer-associated proteins (Table C in S1 Text).

In fact, considering the interactome as a whole, out of the 50 proteins with the highest number of interactions, only six (SRC, EGRF, ESR1, AR, HDAC1 and FYN) are drug targets, all of which are targeted by cancer drugs. This indicates that a large number of first neighbors are not generically associated with being a drug target, but rather the average is skewed by a few highly connected cancer-drug targets.

Network articulation points are nodes that are critical for communication within the network and their removal would disconnect the network into separate graphs or break off peripheral nodes into unconnected singletons. Our analysis shows that 15% of all drug targets, 17% of cancer-drug targets and 14% of non-cancer drug targets are articulation points as compared to 9% of the background set (Table C in S1 Text). However, this enrichment is more statistically significant for all drug targets (p-value = 0.0003) and targets of cancer drugs (p-value = 0.0026) than for targets of non-cancer drugs (p-value = 0.0192). Being an articulation point is akin to an ‘ambassador’ between regions of the network and is a property that is enriched in cancer-drug targets. Interestingly, most articulation points from the cancer-drug target set are through nuclear hormone receptors (NHRs) and receptor tyrosine kinases (RTKs), which are logical gateways for signaling. We also found that cancer targets are more embedded in their local environment than targets from other therapeutic areas (using Burt’s network constraints [13,14,35] and closeness centrality [9,15,36]; Fig C in S1 Text). In summary, our analysis of 28 topological parameters indicates that there are distinguishing patterns of behavior between 1) cancer-drug targets; 2) targets of non-cancer drugs; and 3) the background interactome as a whole. This indicates that the topological parameters can be used as useful features in a predictive model for *ab initio* identification of drug targets for cancer or for non-cancer drugs.

### Cancer targets interact with multiple ‘communities’

A community within a network is defined a set of nodes that are densely connected within subsets of the full interactome (see Methods) but may not all interact directly with each other [16–18,37]. Proteins in a community may be linked together via a function, such as belonging to a particular cellular process. Previous studies have shown the relationship between biological function and network communities [19,37,38]. Drug targets from non-cancer therapeutic areas tend to be members of smaller communities compared to other proteins (Fig C in S1 Text). Interestingly, cancer-associated proteins participate in significantly larger communities, indicating the far-reaching effects of biological malfunctions in this class. Furthermore, cancer-drug targets differ from non-cancer-drug targets when considering their community pattern of interactions. To assess the type of community interactions that a protein is involved in we developed a vertex modularity score based on the proportion of interacting neighbors that are in the same community (see Methods). We find that non-cancer-drug targets tend to interact intra-community, whereas cancer-drug targets, interact both intra- and inter-community. This indicates that while targets of non-cancer drugs address specific functions and defined processes, cancer-drug targets may have wider reaching effects on different cellular functions. This pattern holds equally true for targets of classical cytotoxic cancer drugs, such as tubulin, as for the modern class of cancer genome-targeted cancer drugs, such as kinase inhibitors (Fig D in S1 Text).

### Cancer drug targets disproportionately occupy highly connected graphlets

**Fig 1:**
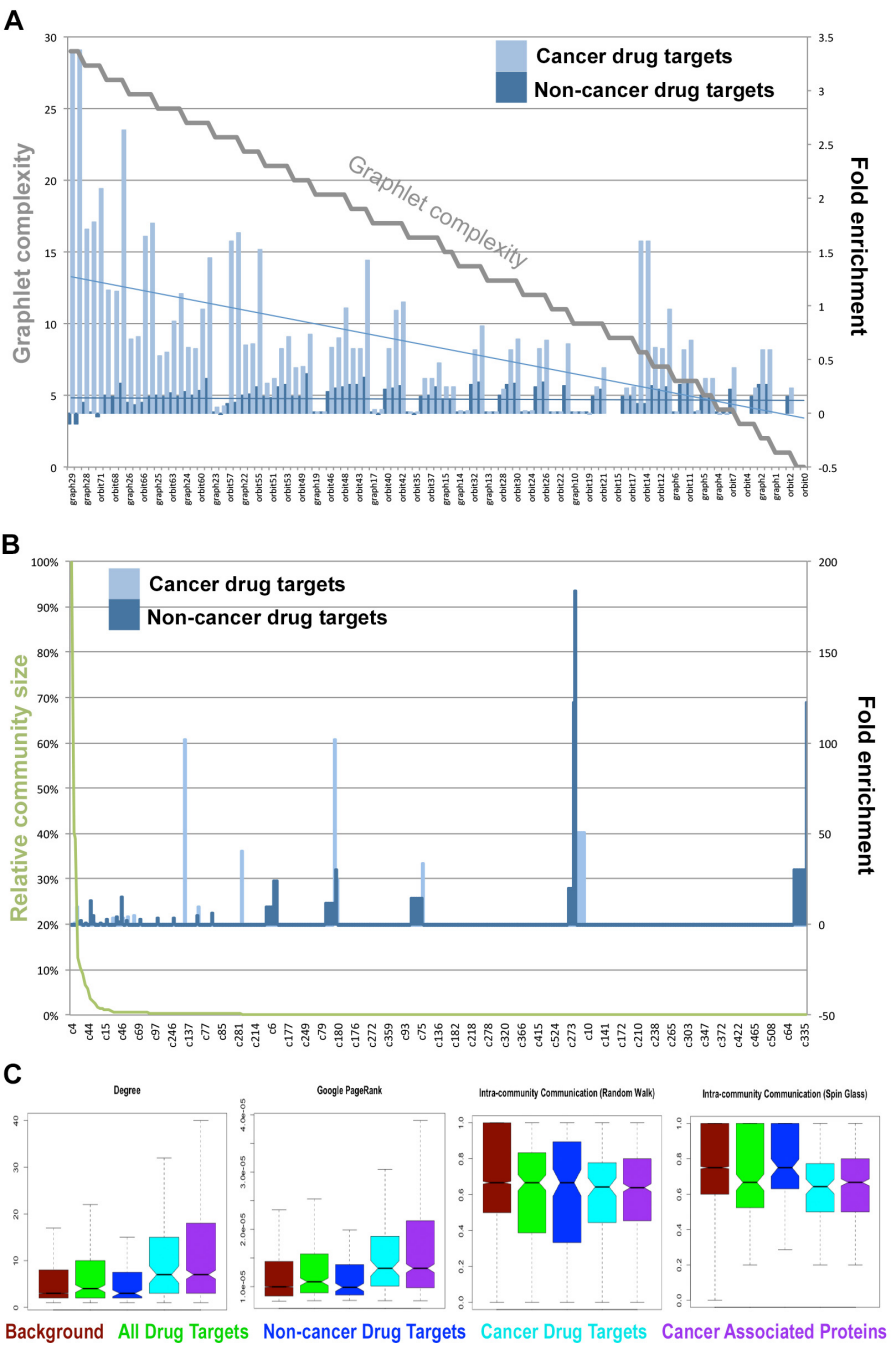
Enrichment and depletion of key parameters in drug targets over what can be expected at random from the interactome. A) Graphlets and their constituent isomorphism orbits. The graph shows the graphlets and orbits, ordered by descending size and complexity, most enriched in cancer-drug targets (light blue bars). These same graphlets and orbits are either slightly depleted or not differentiated from random in targets of non-cancer drugs (dark blue). The gray line represents graphlets size and complexity (high-to-low). B) The distribution of detected community sizes and the enrichment or depletion of cancer drug targets (light blule) versus targets of drugs used to treat other diseases (dark blue). C) Box plots showing distinction of degree and google page rank; as well as the vertex modularity which distinguishes inter- versus intra-community communication of nodes. Further parameters are shown in the Supporting Information.

Complex networks can be divided into smaller sub-graphs, or graphlets, of increasing complexity [39], (see Methods and Fig B in S1 Text). We find a striking difference in the behavior of cancer-drug targets as compared with targets of non-cancer drugs (Fig 1). Not only are cancer-drug targets significantly more active in graphlets (on average involved in 368 million target-graphlet activities compared to 121 million activities for non-cancer targets), but they are also more commonly seen in complex graphlets (such as G26, G27, G28 and G29) than can be expected at random (Fig 1). Importantly, while targets of cancer therapeutics are enriched in these graphlets when normalized against the interactome background, we find that targets of other therapeutic areas are, in contrast, slightly depleted or show little change from background. On average cancer-drug targets have more publications per node than non-cancer drug targets (with 39 versus 11 publications per node) and both sets are better studied than the background interactome (which has an average of 7 publications per node). Approved cancer drugs primarily target a single functional sites on a single type of protein (such as the catalytic site of a kinase and cytochrome P450s or the hormone binding sites on a hormone receptor). Currently most drugs that target heteromeric complexes fall in non-cancer areas (such as the ligand-gated ion channel blockers used to treat disorders of the central nervous system). The graphlet enrichment pattern that we have described here may be due to cancer targets being members of more transient signaling cascades or transcriptional complexes.

### Predictive power is dependent on a large number of features

Thus far, we have discussed some of the parameters that most obviously differentiate the target sets (cancer targets, non-cancer targets, the background interactome). In order to uncover which network features play the most important role we examined the feature contributions for each of the models generated (see S1 File). We find that no single feature is sufficient for discrimination between the target sets. For example, for the GBM models, the range of maximum relative information carried by any one feature was between 4.78% for the all-drug-target model and 5.86% for the non-cancer target model. Similarly, the maximal mean standard error (%MSE) effect of any one feature for the random forest models was 0.02% across all models. Interestingly, although the top features reported by the different models vary, community and graphlet-based features dominate the list of top 20 highest features produced by all the models, while topological features rank lower.

### Prediction accuracy for known drug targets

Of the 343 drug targets in our interactome, 310 (90%) ranked in the top 25% for druggability according to the overall drug target model. This is a 3.6-fold enrichment of drug targets compared with what might be expected if proteins were ranked at random. In addition to the 343 targets of already FDA approved drugs, a further 3,026 currently undrugged proteins in this top-quartile most druggable set (see S1 Table), highlighting them as potentially suitable for drug discovery. The lowest ranking was CYP17A1, the molecular target of abiraterone with a rank of 49%. Additionally, we examined how our network-based assessment of druggability performed in relation to several targets of drugs that are under clinical investigation. We examined targets from different protein families and molecular classes. We found that, despite not having representatives of their families, TLR7, BCL2, EZH2, and MDM2 for example, scored highly using the druggability models (74% or higher using the all-drug-target model; and 85% or higher using the cancer-drug-target model). Full results for seven targets of investigational therapeutics are shown in Table F in S1 Text.

### The effect of different datasets on prediction

To examine the persistence of the signal we investigated the predictive models and target coverage across different datasets. First, we explored the effect of utilizing large-scale Yeast2Hybrid (Y2H) data instead of compiling all high-quality binary interaction data from different sources. Although the Y2H technique is unbiased, we found many interactions were missing from the interactome. This is probably because the full matrix of bait-and-prey proteins has not yet been fully examined. We describe this analysis in detail under the section ‘Defining the interactome’ in S1 Text. In summary, we compiled a large Y2H interactome by collecting Y2H data from 5,537 publications, including 30 publications reporting at least 70 proteins. This resulted in an interactome containing 10,998 proteins and 47,994 interactions–covering 256 of the 345 drug targets in the training set. To complement this we compiled a more comprehensive interactome containing all Y2H studies and high quality data from multiple sources (see Methods). This resulted in an interactome containing 13,345 proteins and 89,691 interactions and covered 343 of the 345 drug targets in the training set. As well as missing 26% of the drug target training set, the large Y2H interactome was missing many known interactions which should have been detected using the methodology. For example, despite MTOR and its complex components such as FKBP1A and DEPTOR being nodes in the Y2H studies, no interactions between them have been reported so far, despite these interactions being experimentally validated outside of Y2H studies [40]. We found that the predictive power of the models is stronger when network properties were calculated using the full interactome rather than the Y2H interactome (See Fig G in S1 Text). We detail all models and prediction results in the Supporting Information.

Additionally, we defined non-redundant versions of the training sets based on protein sequence similarity, drug chemical similarity and therapeutic class similarity (see Methods). Again, we found that models built using these training sets underperformed in comparison with models built on the full training set (Fig H in S1 Text and section ‘Further Information’).

### Druggable nodes and druggable neighborhoods

To identify potential novel drug targets, we collated the top 20 proteins, that are not targets of approved pharmaceuticals and are predicted to be druggable using each of our three models (removing any duplicates). This resulted in a set of 49 proteins shown in S2 Table with their rank from each model and any known link to a disease (using the Online Mendelian Inheritance in Man, OMIM [41] and the Cancer Gene Census [27]). The distinctions in the prediction ranks illustrate the significant differences between drug targets for cancer and drug targets for non-cancer diseases. The 49 proteins fall into 28 protein families. Despite not including any functional or family annotation in the training descriptors, and focusing only on network parameters, we find enrichment in a number of families and classes. The list of 49 proteins contains 18 enzymes, of which six are phosphatases and three are protein kinases. It also contains five G-protein coupled receptors (GPCRs) and five ephrin ligands of receptor tyrosine kinases. Interesting, as well as identifying targets that are druggable, the network-based method additionally identified ligands of drugged or druggable proteins. In summary, the results obtained using our predictive network-based models reflect the enrichment of these druggable target families that is seen in targets of approved pharmaceuticals [32,42].

Additionally, among the 49 top proteins are 18 cell surface proteins and several secreted growth factors (S2 Table). It is interesting that these protein classes are identified as druggable although they are not significantly represented in the training set. However, as the training sets include all targets of FDA-approved drugs, be they targets of small molecules or biotherapeutics, it is reassuring that these cell surface targets are scoring highly as they can potentially be drugged by biotherapeutics such as monoclonal antibodies. Furthermore, at least two of these cell surface or secreted proteins are known ligands of existing drug targets (S2 Table). Similarly, several of the top-scoring proteins are adaptor proteins, three of which are known to interact with existing drug targets. Overall, 23 of the 49 proteins have direct interactions with targets of FDA-approved drugs. Thus the methods seem to identify druggable neighborhoods in the interactome as well as individual druggable nodes. Several of the top-scoring proteins of the whole interactome (S1 Table) are similarly ligands or direct interactors of drug targets, indicating that the predictive models are identifying druggable connections or network neighborhoods and not just individual drug targets.

To compare our network druggability assessment with other methods of scoring druggability, we used the protein annotation tool in canSAR [17,34,42] to obtain 3D structure-based and ligand-based druggability information for our top 49 proteins (S2 Table). Approximately half of them (24 proteins) can be linked to disease using OMIM or the Cancer Gene Census. We found that 31 of the 49 proteins have 3D structures available and, of these, 16 (52%) have at least two independent structures that are predicted to possess druggable cavities [17,34,42,43]. In comparison with the coverage of the proteome for which an estimated 25% is predicted to be druggable by the same criteria [29,30,34,44], this is a 2-fold enrichment in druggability and shows a degree of concordance between the two independent network- and structure-based druggability predictions, without the bias of functional or family annotations. The overlap may increase in the future with improved coverage of 3D structures for the proteome. Twenty four of our top 49 proteins are bound by bioactive small molecules (S2 Table) at sub-micromolar concentrations, according to the medicinal chemistry literature [34,43]. Using the ligand-based chemical druggability score that ranks targets based on the drug-like properties of bioactive compounds [34,42], we find that 28 of the 49 proteins rank in the top 25% most druggable proteins in the proteome showing a 2.3-fold enrichment over what would be expected at random. Again this highlights that the output from our network-based methodology overlaps, and complements, other independent measures of druggability despite using completely different training sets and parameters. Note that many targets cannot be assessed for ligand-based druggability or have low scores because of a lack of available chemical compound bioactivity data; thus the overlap may well increase with time as more targets are chemically explored [42].

### A global view of the druggable interactome

**Fig 2:**
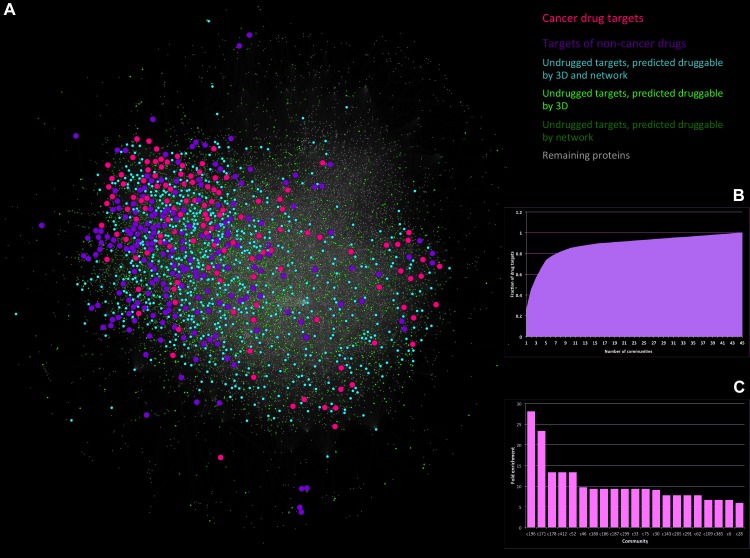
Cancer-drug targets are enriched for highly connected Graphlets. A) Interaction network highlighting the distribution of targets of approved cancer drugs (pink); targets of approved drugs from non-cancer therapeutic areas (blue); and targets predicted to be druggable by different druggability prediction methodologies(light and dark green). Druggable proteins are spread widely across the network while targets of current approved drugs tend to cluster into few areas. B) Cumulative fraction of all drug targets covered by communities. As indicated, a small number of communities cover the majority of drug targets. C) The network communities most enriched in drug targets are listed against the fold enrichment of the number of targets found in them (compared to what can be expected at random).

An annotated, community-correlated map of the human interactome as described in this study is shown in Fig 2A. Although at first glance the targets of FDA-approved drugs (blue and pink) appear widely distributed, detailed inspection shows that they are concentrated in certain areas, often clustered together, whereas non-cancer drug targets are more widely distributed. There are 148 communities of size greater than 4 in this network, yet 70% of all drug targets are in the top 10 communities (Fig 2B). Furthermore, one community shows a 23-fold enrichment in the number of cancer-drug targets that it contains over what would be expected at random (Fig 2C). This probably reflects historical biases where focus was on a few easier-to-drug families or on specific, well-studied disease pathways. However, there are druggable opportunities across most regions of the interactome, as shown by the proteins that are predicted to be druggable using protein 3D structural parameters and the network parameters described in this work. Comparing the output from the three orthogonal predictors of druggability (Network-based, as presented in this method, 3D structure-based and chemical/ligand-based [34]) shows significant overlap despite basing their predictions on completely independent properties (Fig I in S1 Text).

This global view highlights large numbers of potentially missed opportunities and novel target spaces that can be explored, provided that these potential targets are validated for disease causation. Chemical exploration of these barren areas of the interactome, that are predicted to be druggable by both structure- and network-based methodologies, may well yield novel targets for future drug discovery.

**Fig 3:**
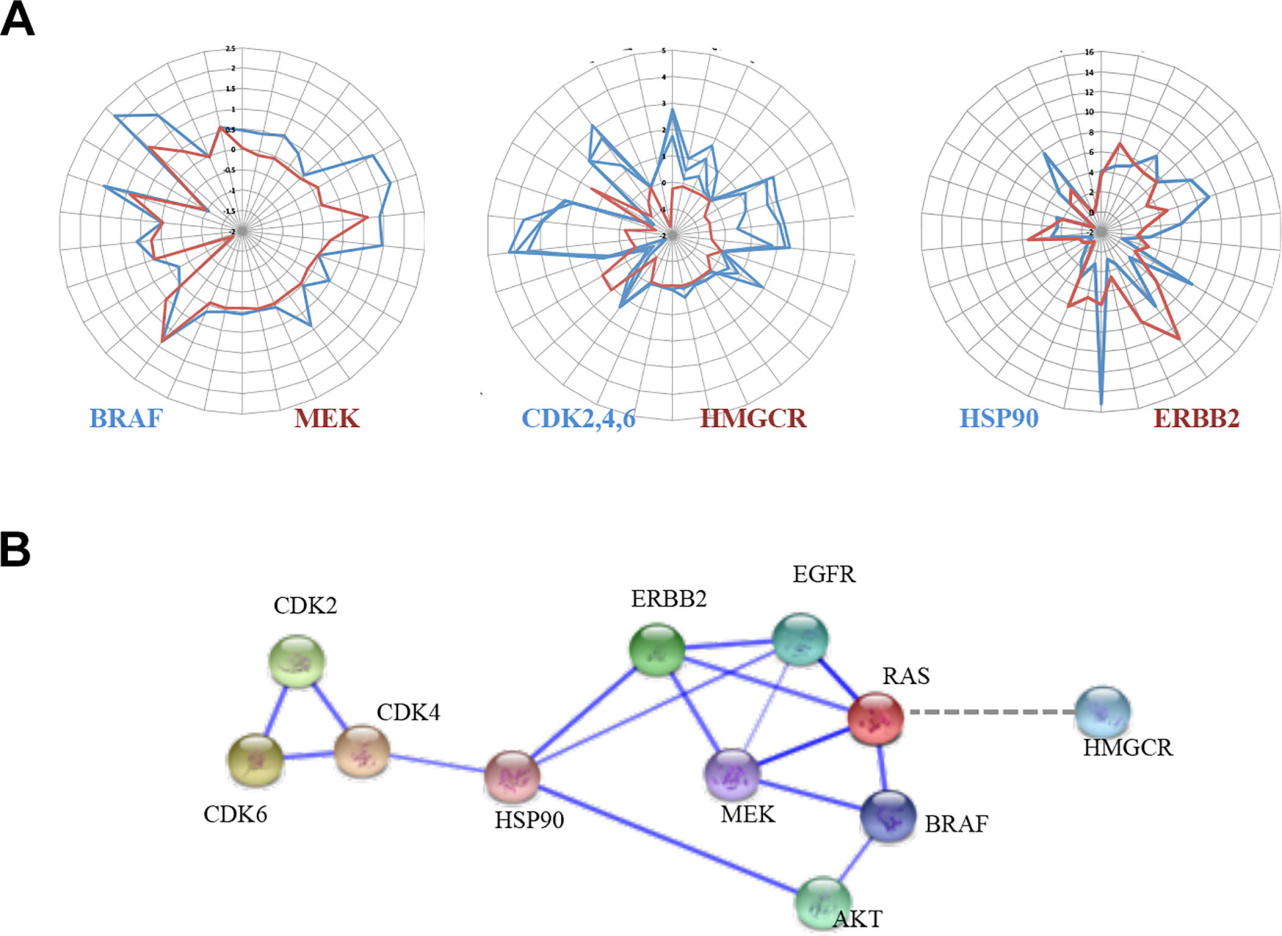
Network profiles and interactions between targets of drug combinations. A) Radar plots showing representative network property profiles of targets of drug combination. MEK and BRAF network property profiles are more similar to one another than the network profiles of CDKs and HMGCR. This may be related to the long-term effectiveness of the combinations of drugs targeting these proteins. B) Interactions between proteins targeted by drug combination showing high level of connectivity between targets such as EGFR, BRAF and MEK. The dotted edge indicates that no direct interaction takes place between HMGCR and the other proteins in the network.

There is a striking difference in the behavior of the cancer-drug versus non-cancer drug targets in the key network parameters described above, such as community behaviors and graphlet structures. This poses an important question: do these, apparently inherent properties of cancer-drug targets, make it easier for the cell to adapt signaling cascades and remodel the network in response to target inhibition? Furthermore, does this contribute to the emergence of drug resistance? Acquired drug resistance through remodeling of signaling pathways is frequently encountered in cancer therapy [30,45] and one possible way of overcoming such resistance may be through the use of combinations of drugs that target proteins occupying different network environments. We compared the network parameter profiles of the targets of well-studied drug combinations (detailed in section ‘Further information’ in S1 Text) using the limited available data. Our analysis suggests that resistance to drug combinations is more likely to occur if they act on targets with similar network profiles, and which are in close proximity in a subnetwork (such as BRAF and MEK [46,47]) compared to drug combinations that act on targets with different network environments [46,48,49] (see Fig 3).

When combining drugs acting on targets with close network proximity, the inhibition of three or more of these targets seems to be required to prevent the emergence of resistance [46]. Despite these intriguing observations, there is insufficient experimental data to allow statistical examination of whether combinations targeting different network environments have a longer-lived effect than those targeting proximal and similar network nodes.

## Discussion

We have presented a systematic, large-scale comparison of 321 topological, community and graphical network parameters for a fully connected interactome of 13,345 proteins and almost 90,000 interactions, totaling 4.2 million calculated properties. We identified significant differences in the network environments that are occupied by cancer-drug targets, non-cancer-drug targets, and the overall interactome. We found a major difference between the degree of cancer-drug targets which tend to have a greater number of first neighbors and be more hub-like, and the degree of non-cancer-drug targets, which, have fewer first neighbors than the interactome average. We found that cancer-drug targets tend to communicate both within and across network communities unlike non-cancer-drug targets that primarily communicate within their communities. Overall, community behavior and subgraph connectivities played the most significant roles in this distinction. Indeed it takes a complex interplay of topological, graphical and community behaviors to provide discriminatory signatures that can distinguish cancer-drug targets from non-cancer-drug targets and from the interactome as a whole. These signatures led to the generation of predictive models that predict druggable network nodes and neighborhoods with an average accuracy of 83%.

As well as identifying targets of approved drugs, the network druggability prediction models described here identified both potentially druggable targets and target local neighborhoods, providing an independent and complementary method of assessing the suitability of a target for therapeutic modulation. The methods presented in this study use only network parameters and the training sets include targets of all approved therapeutics and not just small molecule drugs. Despite this, the output of our network models showed strong concordance with the output from other orthogonal methods that use 3D structural information or ligand binding data to predict druggability. To enable the research community to use our methodologies for objective and independent target prioritization, we have provided the results of our network-based predictions alongside structure-based and ligand-based druggability results within the canSAR website (https://cansar.icr.ac.uk). These models are already useful predictive tools, the predictive power of which can only improve with the elucidation of the full human interactome and the mapping of disease-specific temporal interactions.

Exploration of the network parameters of targets in several examples of resistance to cancer drugs and mechanisms for synergistic drug targets suggests that the combination of modulators of distinct environments within the cell may be a more effective approach to overcome drug resistance than modulating targets with similar network environments. As more data from systematic, large-scale drug combination screens and clinical practice becomes available, we will be able to explore the extent to which such predictions of effective drug combinations are useful and if the can provide us with an a priori systems view of selected therapies.

The global view of the interactome presented here provides insights into important, but often neglected, systems-based considerations that should be included when selecting a target for therapeutic investigation which have the potential to inform better drug combinations.

## Methods

### Defining the ‘interactome’

Data imbalance, redundancy and lack of clear quality measures are all problems in defining the human interactome [19,31]. The ideal solution would be the availability of a comprehensive and unbiased protein-interaction data collection. Data from Yeast-2-Hybrid (Y2H) studies (e.g. [50,51]) are making headway towards this goal, yet currently only cover a fraction of the human interactome (detailed in ‘Further information’ in S1 Text). Nonetheless, for objective comparison, we created three separate views of the human interactome: Set A) comprising only published Y2H studies from large-scale Y2H publications containing at least 1000 proteins–this interactome contains 7,722 proteins and 24,406 interactions; Set B) all Y2H data that we could identify in the public domain–this utilized 5,537 publications and includes 10,998 proteins and 47,994 interactions; and Set C) the full experimental interactome including all Y2H publications as well as other high quality interaction data. For the Set C interactome we collected the human protein-protein interaction data from the partners of the International Molecular Exchange Consortium (IMEx [19]), Phosphosite (http://www.phosphosite.org/), and structurally-determined complexes from the Protein Data Bank [52]. We removed ambiguous interactions derived from converting a protein complex into a set of binary interactions. We created a network using R igraph package [53]. In order to compensate for the differing amounts of interactions between the proteins, we removed isolated proteins and isolated small subnetworks (Fig A in S1 Text). This resulted in a single network consisting of 13,345 proteins with 89,691 interactions and no unconnected nodes or networks. Despite our stringency in data selection, this network still contains roughly 66% of the proteome and 37% of the total predicted interactome [54].

### Training sets

We have defined a number of target/protein classes. Firstly, the positive ‘drug target list’ is a list of manually-curated targets of FDA-approved pharmaceuticals [32,34], defined using strict criteria based on known pharmacological action and drug approval information. Thus it is strictly confined to the curated efficacy targets of the drugs rather than targets that may bind a drug without therapeutic effect. The ‘drug target’ list includes targets of both small molecule drugs and biotherapeutics. A total of 343 human drug targets were successfully mapped to the network: of these, 127 are targets of cancer therapeutics, constituting the ‘cancer target list’, while the remainder comprise the ‘drug targets, other therapeutic areas (TA)’ list (Fig A in S1 Text). Finally, we also define a fourth ‘cancer-associated’ protein list, containing proteins that contribute to the pathology of cancer, to be a superset of the cancer-drug targets and protein products of genes from the Cancer Gene Census [27]. Thus 633 of the proteins in the network are labeled as ‘cancer-associated’. For each of the four defined positive sets (e.g. all drug targets) a matching ‘background’ dataset was defined as the remainder of the 13,345 proteins in this study (Table A in S1 Text). To address the bias caused by the correlation between the degree, or number of first neighbors, and other topological descriptors (e.g. hub-score), we further classified the datasets into three categories [17] depending on the number of first neighbors: low (≤5), medium (6–30) or high (≥31) degree (Table A in S1 Text). We examined each of the network descriptors analyzed for the full datasets as well as for these, degree-dependent subclasses of each dataset.

Additionally, we created non-redundant representatives of the training sets: 1) we clustered targets based on sequence similarity using a sequence identify cut-off of 50%; BLAST [55] E-value ≤10^−6^ and at least 30% sequence overlap reducing the drug target training set from 343 to 246 targets, 2) we clustered targets based on the Anatomical Therapeutic Chemical Classification System (ATC) level-3 therapeutic/pharmacological subgroups, reducing the drug target training set to 82, and 3) we clustered the targets based on shared chemical scaffolds using Bemis and Murcko [56] framework definitions; this reduced the target set to 283.

### Network descriptor calculations

We calculated a total of 321 properties that fell into three categories: topological, graph-based and community based features (detailed in Table B in S1 Text). We calculated 31 global- and local-network topological parameters using the igraph package [53] and the Disconnectivity Valuation tool DiVa [57]. We also calculated the Dice similarity coefficient [53] based on fractions of shared neighbors which we converted to a distance matrix and performed multidimensional scaling. We used the two primary dimensions V1 and V2 as part of our topological descriptor set. For community detection, we applied two types of algorithms: Random Walk [58] and Spin-Glass [59] as implemented in igraph. The function walktrap.community was applied with a random walk of length = 4 and spinglass.community was applied with a predefined number of communities set to 50. We developed a bespoke measure of protein community communication behavior, the vertex modularity (VM) computed as the number of a protein's neighbors that are in the same community divided by the total number of neighbors a protein has. Therefore, a high VM number means the protein’s neighbors are in the same community and therefore the protein favors intra-community communication while a low VM number indicates the protein favors inter-community communication. We used GraphCrunch [60] to calculate subgraphs previously described as a means of fragmenting networks into smaller graphlets [39] (Fig B in S1 Text). The nodes within these graphlets can be classified into ‘isomorphism orbits’ [39] (here referred to simply as ‘orbits’), that reflect the pattern of interactions within the graphlet.

### Training sets and predictive modelling

We created four training datasets comprising the positive and background sets (Fig A in S1 Text): drug targets (all TAs), cancer-drug targets, non-cancer-drug targets, and cancer-disease associated proteins. We further split each of these sets into four degree-based subsets as described earlier (all, high degree, medium degree and low degree). This resulted in 16 datasets for modeling (Table A in S1 Text). It is important to note that some of the subsets are very small, such as the ‘Low’ cancer drug target set which contains only 16 proteins and the ‘High’ non-cancer drug targets which contains 23 proteins. These sets are too small for effective model building. We inputted the 321 descriptors, calculated for each of the 16 sets, into three distinct predictive modeling algorithms: Random Forests [61], Gradient Boosted Machines (GBM [62]) and Generalized Linear Models (GLM [63]). Since we can only label proteins as drug targets or background or unlabeled proteins (i.e. it is not possible to assign a negative training set as it is not possible to say which proteins are currently are not drugged but may become successful drug targets in future) we apply a positive-unlabeled (PU) learning paradigm (see e.g.[64]).

All models were implemented in R and the code with the specific algorithm parameters is provided at: https://cansar.icr.ac.uk/cansar/publications/druggable_network_neighbourhoods/

Using the data derived above, we constructed several models to predict: 1) general druggability (the likelihood of a protein to be a drug target for any therapeutic area); 2) cancer druggability (the likelihood of a protein to be a cancer-drug target); 2) non-cancer druggablity (the likelihood of a protein to be a drug target for a non-cancer therapeutic area); and finally 4) cancer-association (the likelihood of a protein to be a cancer-associated protein). Table 1 reports the results of a 10-fold cross validation for the All, Low and Medium datasets and a 5-fold cross validation for the High dataset due to the small minority class. In total, we built 450 models.

**Table 1:** Results of the 10-fold cross-validation of predictive models.

Dataset	Subset based on degree (number of first neighbors)	AUC
Drug Targets	All	83.12
	Low (< = 5)	58.98
	Medium (> = 6, < = 30)	77.51
	High (> = 31)	78.22
Drug Targets (not cancer)	All	80.46
	Low (< = 5)	71.66
	Medium (> = 6, < = 30)	77.97
	High (> = 31)	69.29
Cancer Drug Targets	All	86.44
	Low (< = 5)	NaN*
	Medium (> = 6, < = 30)	75.16
	High (> = 31)	84.97
Cancer-associated	All	82.64
	Low (< = 5)	63.46
	Medium (> = 6, < = 30)	71.85
	High (> = 31)	72.85
Random	All	48.44
	Low (< = 5)	49.80
	Medium (> = 6, < = 30)	43.89
	High (> = 31)	57.29

* The ‘Low’ subset for cancer drug targets contains too few members for a successful 10- or 5-fold cross-validation.

The ‘Random’ class is where the ‘drug target’ classification was assigned to random nodes within the network to allow fair assessment of the models performance without changing the underlying network structure.

Predictive modeling of network data poses an interesting problem when it comes to training the model. The standard is to report the results of a k-fold cross-validation (CV). For example in 10-fold CV, the data are split into training and validation sets and the model is built using the 90% training subset and validated on the 10% subset. This process is repeated 10 times and the average accuracy of the validation is reported as the prediction accuracy. This method is widely adopted as it approximates how the model will perform on new, unseen data. However, with a network, each instance is dependent on other instances as the descriptors are based on the instance’s position in the network. Consequently, using a holdout-set is nonsensical, as there can be no new cases without generating the network data again. To overcome this problem with the 10-fold CV, we recreated random training sets that maintained the structure of the network and the number of positives, but where the positives were allocated to random proteins. We carried out a 10-fold CV on these random sets to compare to the predictive results observed from the true training sets. Another problem for the predictive modeling of the network was the imbalance of the data. The minority classes ranged from 1% to 5% and therefore regression models were built rather than 2-class classification models. As our data comprises only PU data sets, we report the results based on a ranked evaluation of area under the curve (AUC). We ranked the predictions according to their average regression output and calculated the percentile, for example, a score of 78% means that 78% of proteins had a lower rank than this protein.

### Supporting information

There are five supplementary files provided with this document and also on the canSAR website: https://cansar.icr.ac.uk/cansar/publications/druggable_network_neighbourhoods/

## Supporting Information

S1 Text Additional supplementary text and explanation of methodology, together with supplementary data tables and Figs referred to in the main document. (DOCX)

S1 Table The full annotation and network-based druggability predictions of the 13,345 proteins in this analysis (using the largest interactome). The full prediction results for 10,998 proteins using the largest Y2H-based models; model quality and AUCs for the Y2H models.
(XLSX)

S2 Table Details of the top most druggable proteins identified using a network-based druggability analysis that are not themselves targets of FDA-approved drugs. (XLSX)

S1 File Individual predictive results and relative information content of each of the topological, community and graphical features used to train the models. (ZIP)

S2 File File containing the raw data used to generate the correlation plot in Fig F in S1 Text showing the limited correlation observed between the network topological, graphical and community-based features used in our analysis. (XLSX)

S3 File P-values of association between individual features of the drug target classes, namely all drug targets, targets of cancer drugs, or targets of drugs used in other therapeutic areas. (XLSX)
