# Supplementary material for: Distinctive Behaviors of Druggable Proteins in Cellular Networks
Source: PLoS Comput Biol. 2015 Dec 23;11(12):e1004597. doi: 10.1371/journal.pcbi.1004597 (PMC4689399; doi:10.1371/journal.pcbi.1004597)
Supplement: S1 Text — (DOCX) [file pcbi.1004597.s001.docx]

Distinctive behaviors of druggable proteins in cellular networks

Costas Mitsopoulos^1†,^ Amanda C. Schierz^1,2†^, Paul Workman^1^ and Bissan Al-Lazikani^1^*

**Supplementary Information contents**

Figures 3

Figure A: Flowchart representing the steps of the study. Details in the Materials and methods section 3

Figure B: Graphlets 4

Figure C: Boxplots showing the distributions of the most discriminatory topological and community-based parameters that showed difference across all datasets. 5

Figure D: Vertex modularity of targets of cytotoxic drugs versus other targets 6

Figure E: Recall response curve for the full models 7

Figure F: Feature correlations 8

Figure G: Recall response curves for models built using the Y2H interactome data 9

Figure H: Overlaps and Recall response curves for models built from non-redundant sets 10

Figure I: Comparison of predicted druggability using three orthogonal methods 12

Figure J: Difference of Topological parameters between full interactome (Set C) and the large Y2H interactome (Set B). 13

Tables 14

Table A: Datasets 14

Table B: Data dictionary 14

Table C: Datasets, their background equivalents and enrichment of some key topological parameters. 15

Table D: The top 49 proteins predicted druggable using at least one of the three network-based druggability models and that are not themselves targets of approved drugs 16

Table E: Full predictions for 13345 proteins 16

Table F: Network-based druggability of several targets of investigational drugs 16

Further Information 17

Defining the interactome 17

Features used in model construction 20

Validation of predictive models 21

Predictive power of individual features derived from LASSO and EN models 26

Drug Combination Studies 26

References 27

# Figures

## Figure A: Flowchart representing the steps of the study. Details in the Materials and methods section


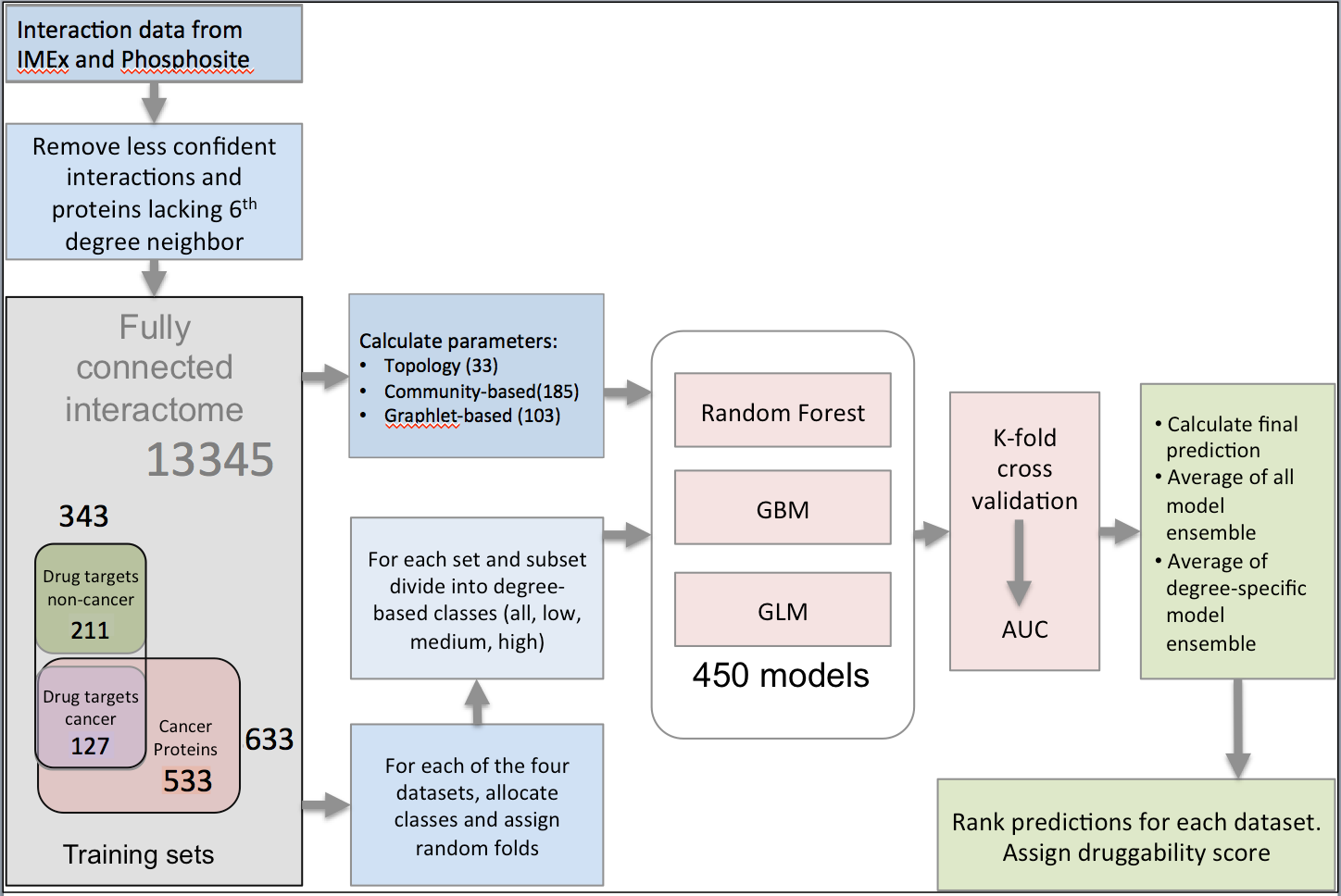


## Figure B: Graphlets

Graphlets and auto-morphism orbits reproduced with permission from [1]


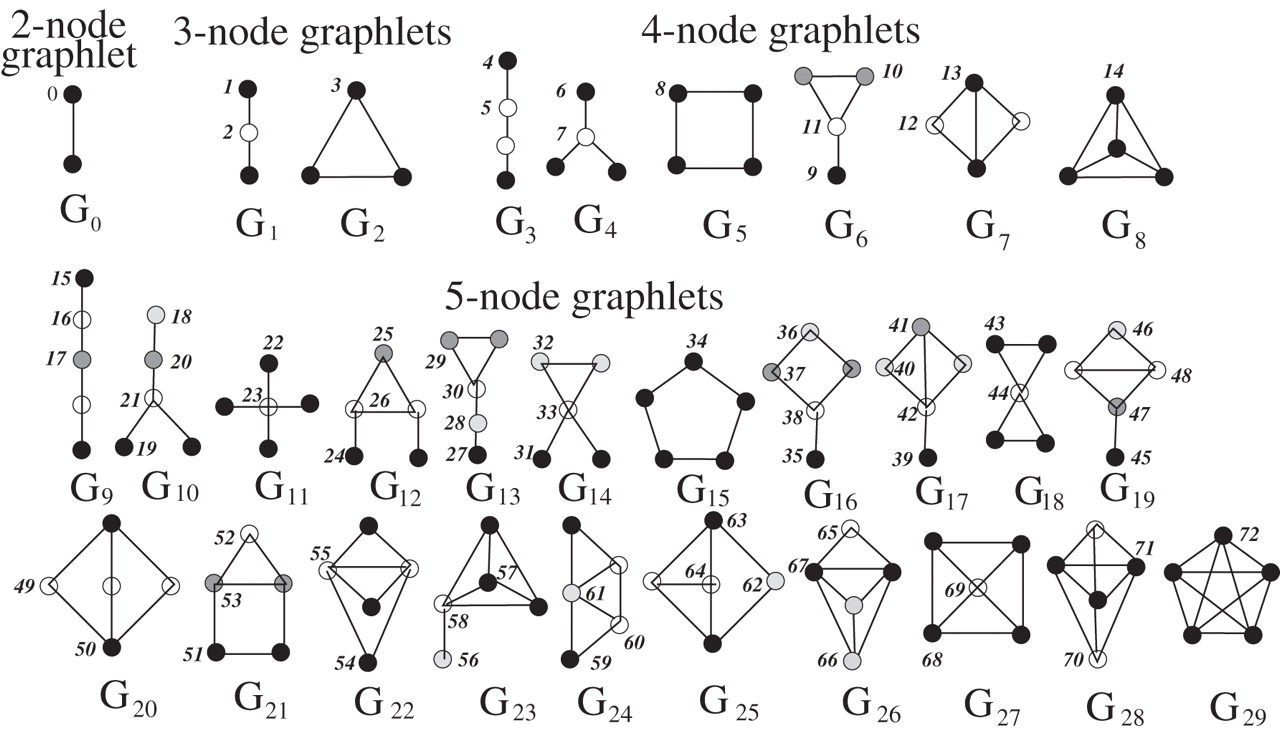


**Definitions**

**Graphlet:**a graphical subset of the network denoting proximal connected nodes. A graphlet can be made up of two or more nodes. One node can belong to more than one type of graphlet.

**Isomorphism orbit, or ‘orbit’:** is a node with a defined connectivity pattern within a graphlet. E.g. Graphlet G_0_ has only one type of orbit.

**Elongated graphlet:** A simple, linear or semi-linear graphlet such as G_9_ and G_10_.

**Complex graphlet:** a highly connected graphlet e.g. G_29_.

**Target activity in Graphlet:** The number of different graphlets that a target node is found in.

## Figure C: Boxplots showing the distributions of the most discriminatory topological and community-based parameters that showed difference across all datasets.


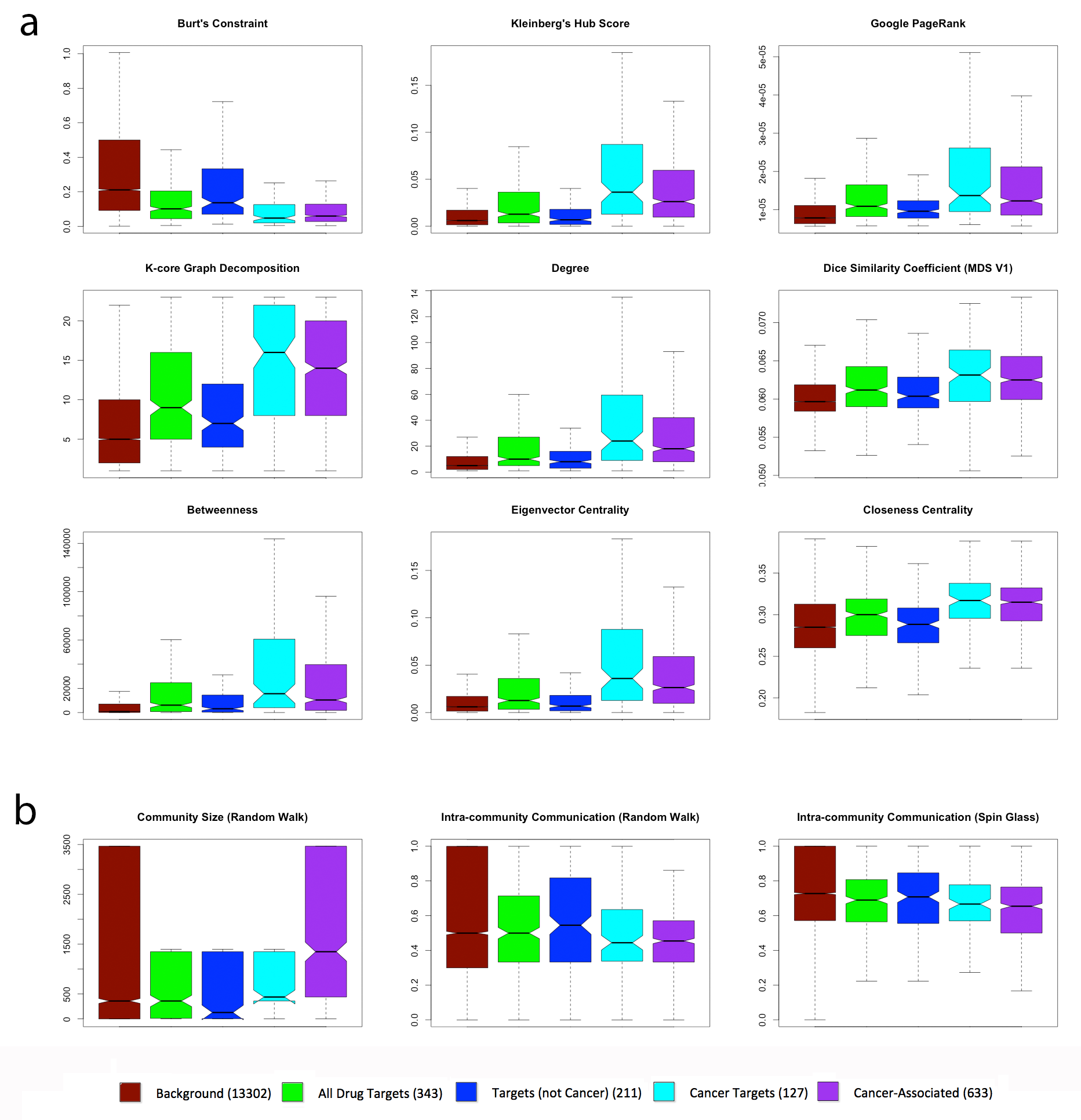


## Figure D: Vertex modularity of targets of cytotoxic drugs versus other targets

Box plots showing the distribution of vertex modularity values (calculated using communities derived from Walk-trap on the left and Spinglass on the right) for targets of cytotoxic drugs (C, n=16), protein kinases (K, n=42) and all other cancer targets (o, n=50). No significant difference in the distributions is observed indicating that the patterns of intra- versus inter-community communications are similar for all these classes of cancer drug targets.

##
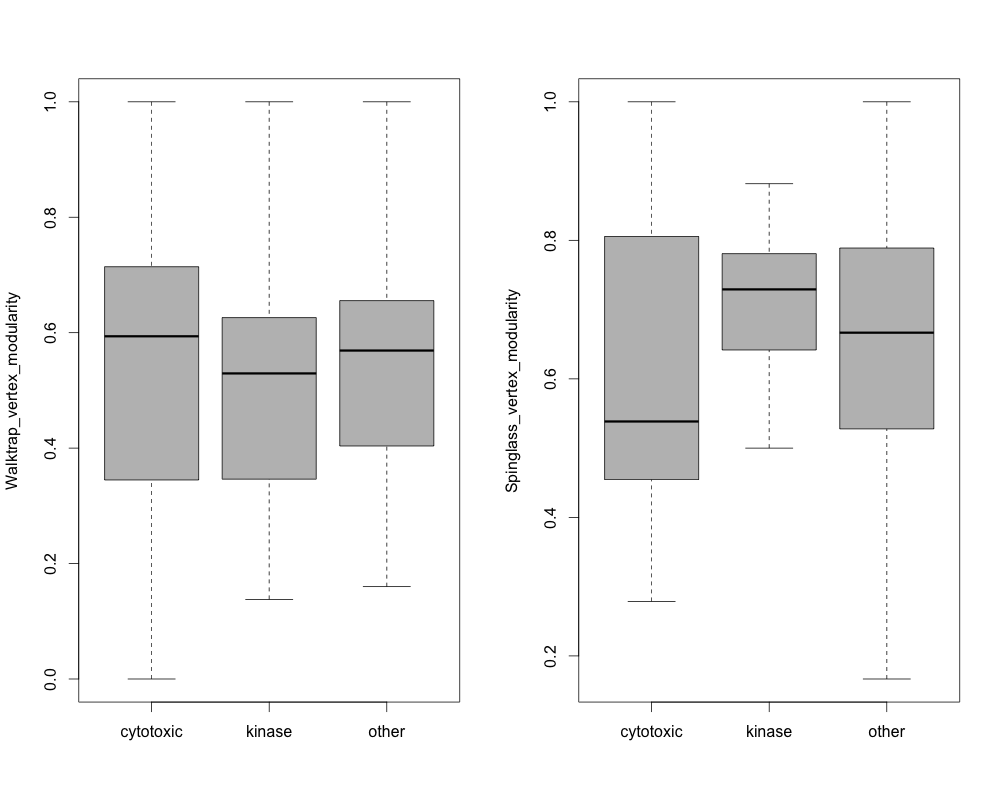


## Figure E: Recall response curve for the full models


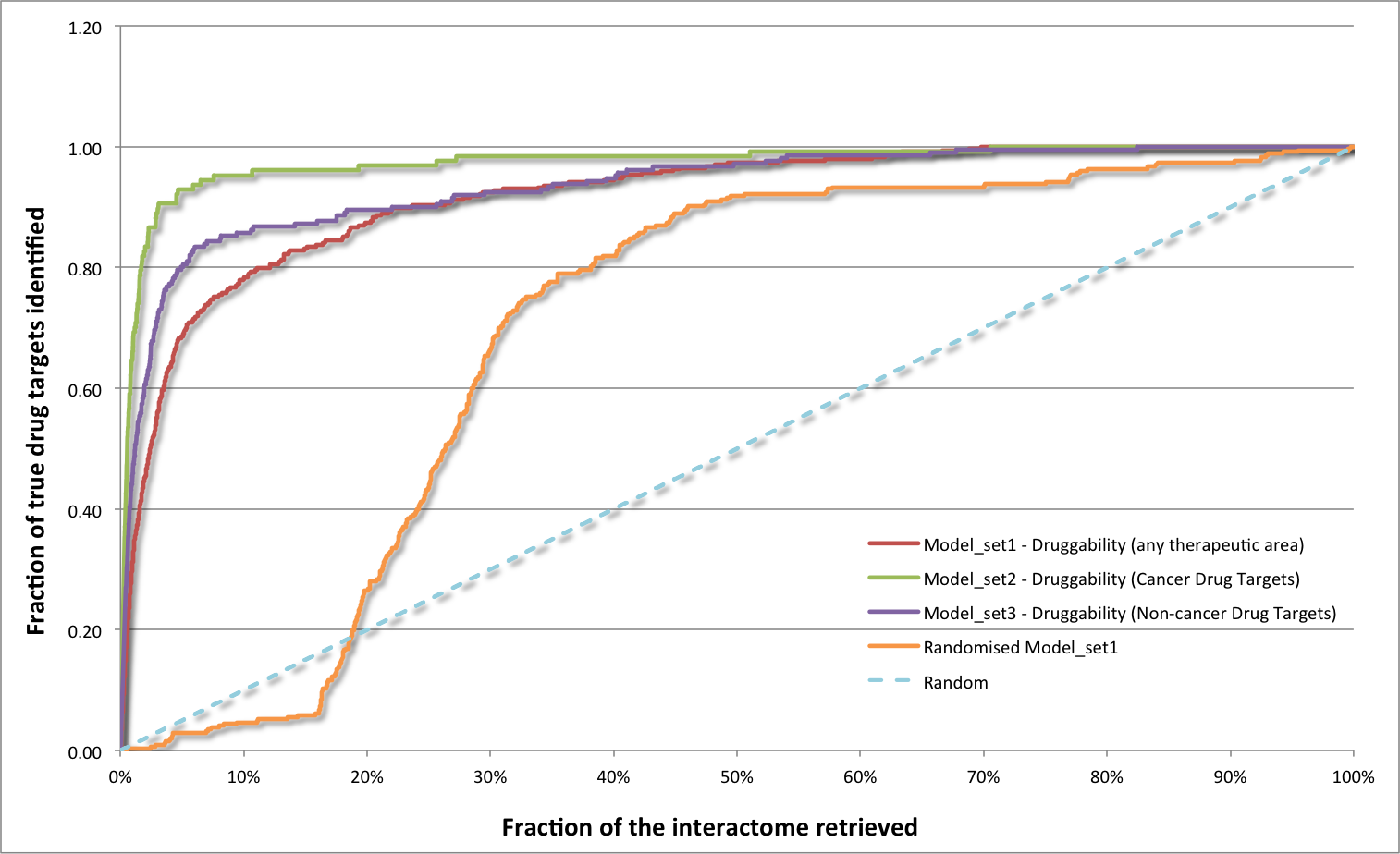


## Figure F: Feature correlations

a)


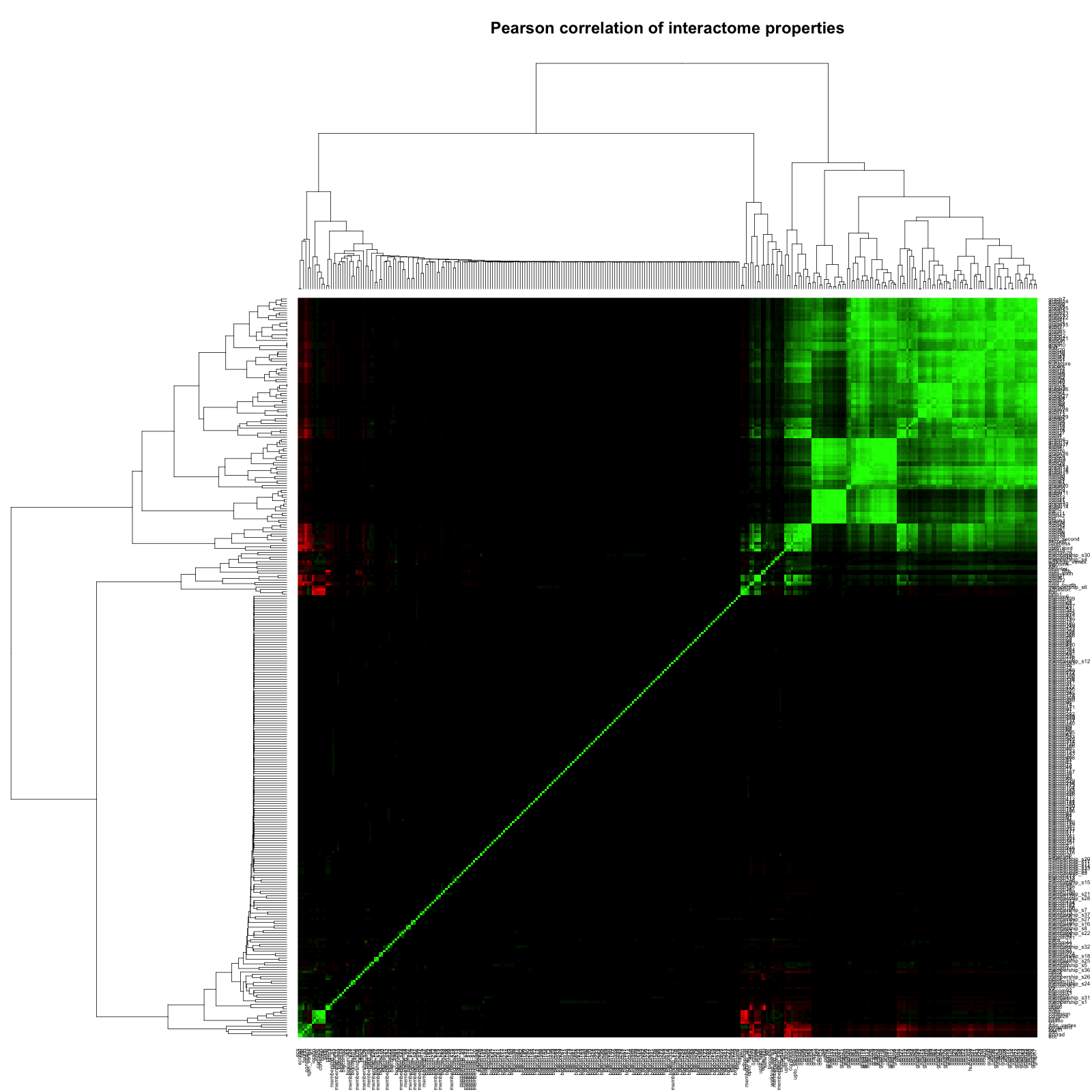


b)


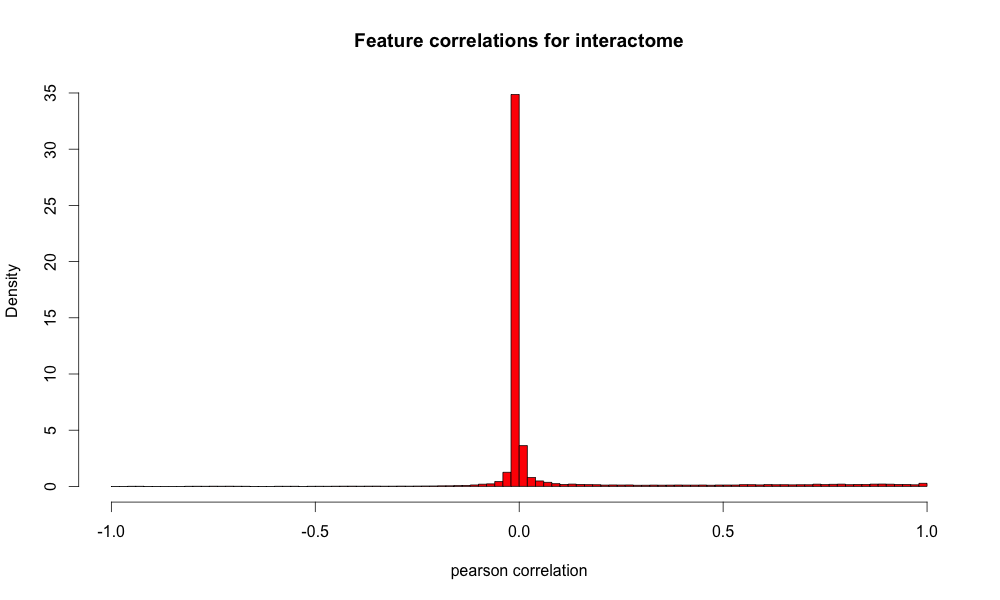


## Figure G: Recall response curves for models built using the Y2H interactome data

## Figure H: Overlaps and Recall response curves for models built from non-redundant sets

**
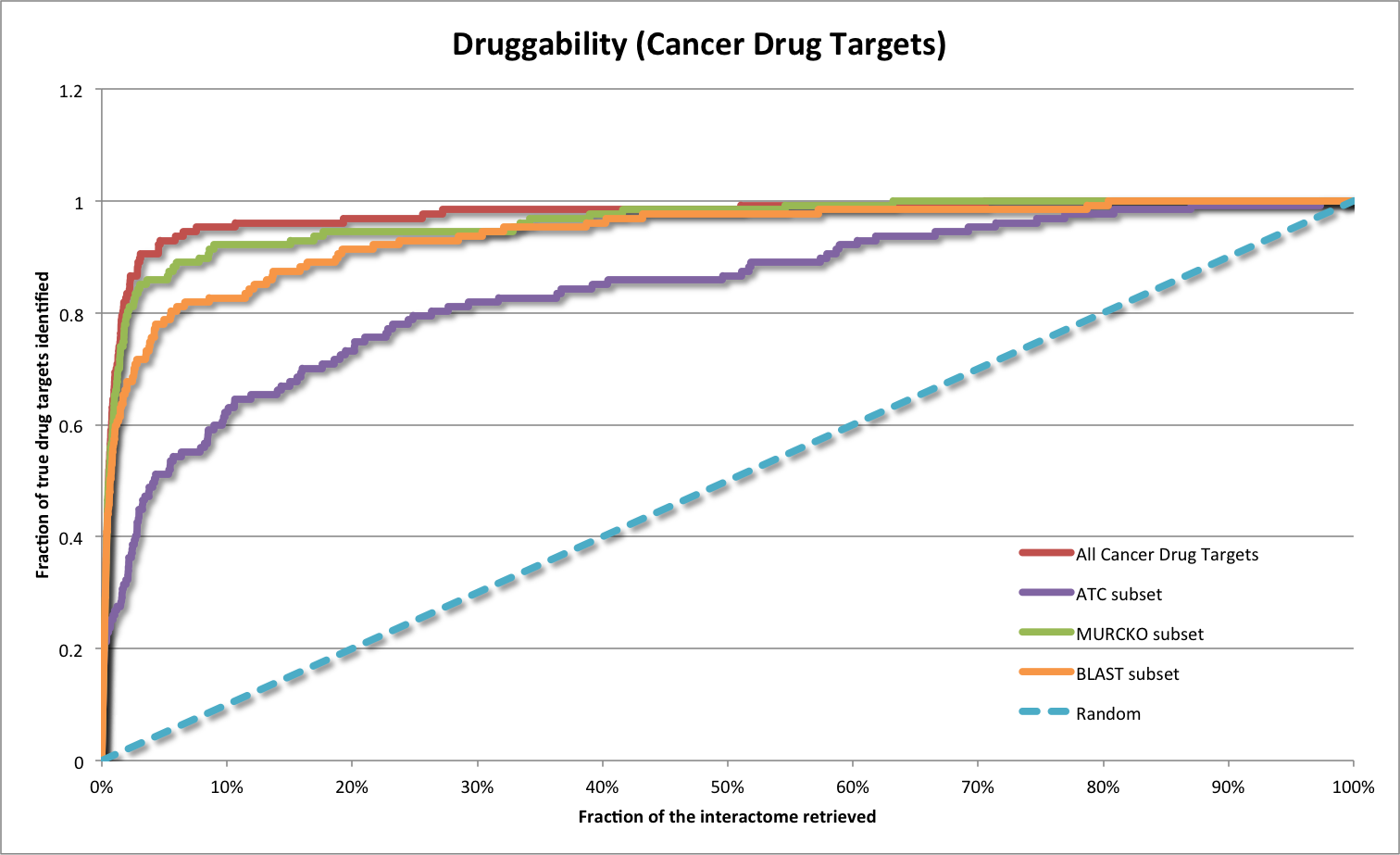
**


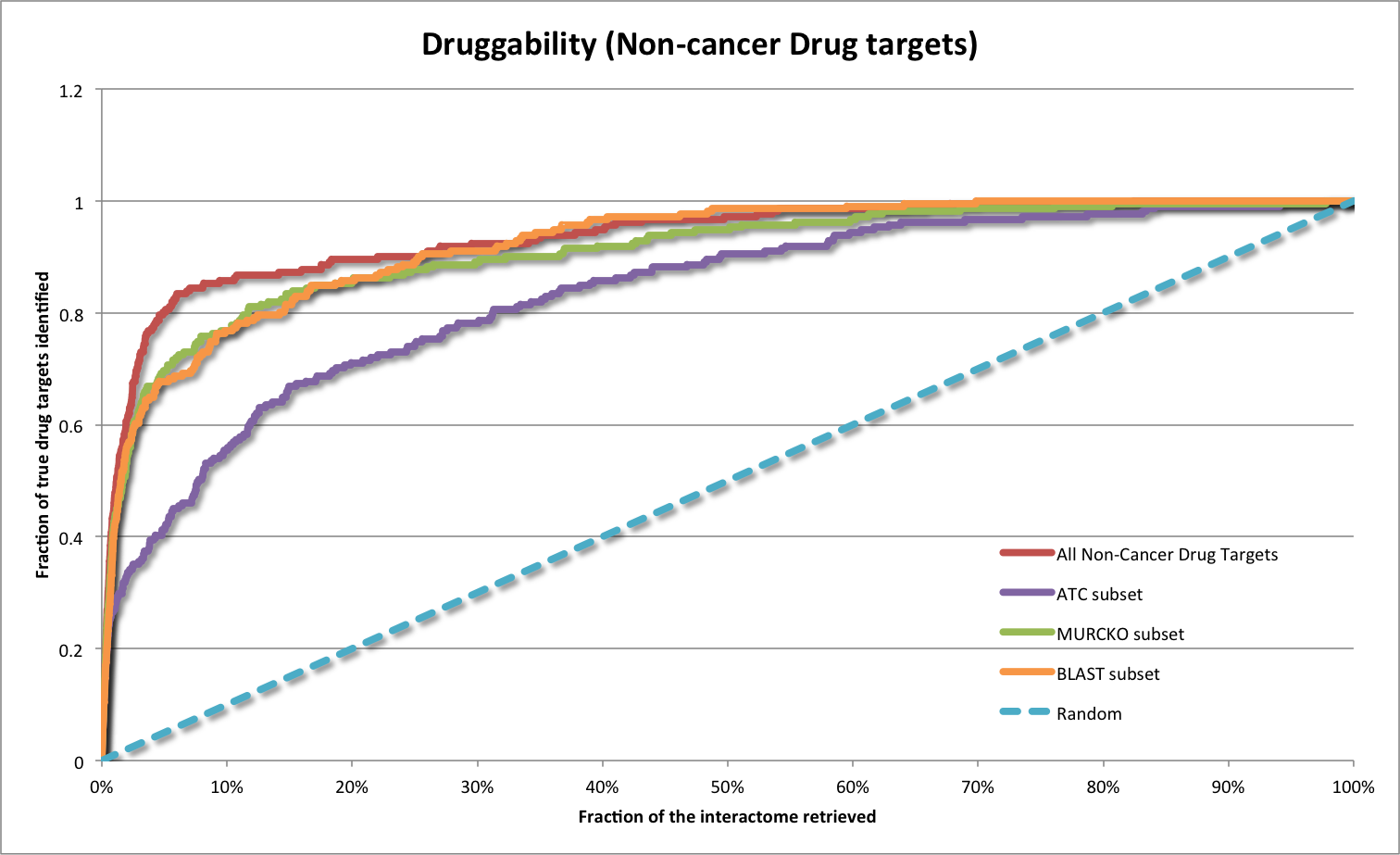


## Figure I: Comparison of predicted druggability using three orthogonal methods

## Figure J: Difference of Topological parameters between full interactome (Set C) and the large Y2H interactome (Set B).

# Tables

## Table A: Datasets

| **Dataset** | **Neighbours** | **Total** | **Positive** | **Negative** |
| --- | --- | --- | --- | --- |
| Drug Targets | All | 13345 | 343 | 13002 |
| Drug Targets | Low (<=5) | 6968 | 95 | 6873 |
| Drug Targets | Medium (>=6, <=30) | 5128 | 174 | 4954 |
| Drug Targets | High (>=31) | 1249 | 74 | 1175 |
| Drug Targets (not cancer) | All | 13345 | 211 | 13134 |
| Drug Targets (not cancer) | Low (<=5) | 6968 | 77 | 6891 |
| Drug Targets (not cancer) | Medium (>=6, <=30) | 5128 | 111 | 5017 |
| Drug Targets (not cancer) | High (>=31) | 1249 | 23 | 1226 |
| Cancer Drug Targets | All | 13345 | 127 | 13218 |
| Cancer Drug Targets | Low (<=5) | 6968 | 16 | 6952 |
| Cancer Drug Targets | Medium (>=6, <=30) | 5128 | 61 | 5067 |
| Cancer Drug Targets | High (>=31) | 1249 | 50 | 1199 |
| Cancer-associated | All | 13345 | 633 | 12712 |
| Cancer-associated | Low (<=5) | 6968 | 101 | 6867 |
| Cancer-associated | Medium (>=6, <=30) | 5128 | 320 | 4808 |
| Cancer-associated | High (>=31) | 1249 | 212 | 1037 |

## Table B: Data dictionary

| **Data Class** | **Description** | **Quantity** | **Data Type** |
| --- | --- | --- | --- |
| Graphlet Degree Distribution | Graphlets | 30 | Integer |
| Graphlet Degree Distribution | Orbits | 73 | Integer |
| Community-based | Walktrap communities | 148 | Boolean |
| Community-based | Spin-Glass communities | 29 | Boolean |
| Community-based | Inner links | 1 | Integer |
| Community-based | Outer links | 1 | Integer |
| Community-based | Inner/Outer ratio | 1 | Continuous |
| Community-based | Community Size | 1 | Integer |
| Community-based | Adhesion | 1 | Continuous |
| Community-based | Cohesion | 1 | Continuous |
| Community-based | Neighbours/Community ratio | 2 | Continuous |
| Topological | Number of 1^st^ to 6^th^ neighbours | 6 | Integer |
| Topological | Cumulative number of 2^st^ to 6^th^ neighbours | 5 | Integer |
| Topological | Ratios of neighbours | 6 | Continuous |
| Topological | Eccentricity | 1 | Integer |
| Topological | Eccentricity/Radius ratio | 1 | Continuous |
| Topological | Betweenness | 1 | Continuous |
| Topological | Closeness Centrality | 1 | Continuous |
| Topological | Burt’s Constraint | 1 | Continuous |
| Topological | k-core | 1 | Integer |
| Topological | knn nearest neighbour degree | 1 | Continuous |
| Topological | Kleinberg Hubscore | 1 | Continuous |
| Topological | Google PageRank | 1 | Continuous |
| Topological | Transitivity,Clustering Coefficient | 1 | Continuous |
| Topological | Self-Interacting | 1 | Boolean |
| Topological | Pairwise Disconnectivity Index | 1 | Continuous |
| Topological | Eigenvector Centrality | 1 | Continuous |
| Topological | MDS 1 and 2 | 2 | Continuous |
| Topological | Articulation Point | 1 | Boolean |

## Table C: Datasets, their background equivalents and enrichment of some key topological parameters.

| **Dataset** | **Positive/**  **Background** | **Number of proteins** | **Mean Degree** | **Median Degree** | **Articulation Points (AP)** | **Self-Interacting (SI)** |
| --- | --- | --- | --- | --- | --- | --- |
| All |  | 13345 | 13.00 | 5 | 1271 (10%) | 2926 (22%) |
| Drug Targets (DT) | + | 343 | 26.34 | 10 | 52 (15%) | 165 (48%) |
|  | - | 13002 | 12.65 | 5 | 1219 (9%) | 2761 (21%) |
|  | *p-value* | | *1.69E-15**** |  | *0.00031*** | *1.15E-32**** |
| Drug Targets  (non-cancer) (DTNC) | + | 211 | 13.72 | 8 | 30 (14%) | 82 (39%) |
|  | - | 13134 | 12.99 | 5 | 1241 (9%) | 2844 (22%) |
|  | *p-value* | | *0.73699* |  | *0.01921* | *2.01E-09**** |
| Cancer Drug Targets (CDT) | + | 127 | 47.21 | 24 | 22 (17%) | 81 (64%) |
|  | - | 13218 | 12.67 | 5 | 1249 (9%) | 2845 (22%) |
|  | *p-value* | | *5.15E-35**** |  | *0.00262** | *1.63E-30**** |
| Cancer associated (CA) | + | 633 | 36.53 | 18 | 105 (17% | 283 (45%) |
|  | - | 12712 | 11.83 | 5 | 1166 (9%) | 2643 (21%) |
|  | *p-value* | | *5.82E-84**** |  | *5.41E-10**** | *4.68E-46**** |

## Table D: The top 49 proteins predicted druggable using at least one of the three network-based druggability models and that are not themselves targets of approved drugs

See separate excel spread sheet entitled S2_Table.xlsx

## Table E: Full predictions for 13345 proteins

See separate excel spread sheet entitled S1_Table.xlsx

This additionally includes the full prediction results for 10,998 proteins using the largest Y2H-based models; model quality and AUCs for the Y2H models.

## Table F: Network-based druggability of several targets of investigational drugs

| **Target** | **Score (alldrug targets)** | **Score (Cancer Drug Targets)** | **Score (Non-cancer Drug Targets)** |
| --- | --- | --- | --- |
| BCL2 | 93% | 99% | 79% |
| TLR7 | 96% | 85% | 99% |
| EZH2 | 75% | 94% | 65% |
| BRD4 | 55% | 75% | 47% |
| TNKS | 78% | 77% | 59% |
| PIK3CA | 92% | 91% | 94% |
| MDM2 | 74% | 98% | 92% |

# Further Information

## Defining the interactome

In defining the interactome for this analysis we attempted to balance the size and completeness of the interaction data with its quality as described in the methods. Data imbalance is a major problem in defining the human interactome. Some proteins may appear to have a large number of interactions simply because they have been well-studied, while others may appear to have only a small number of interactions because of lack of experimental investigation.

The only comprehensive way to properly address this imbalance problem is to use an interactome that was obtained using unbiased experimental techniques such as Yeast-2-Hybrid (Y2H). Data from Yeast-2-Hybrid (Y2H) studies (e.g. [2,3]) are making headway towards this goal, yet currently only cover a fraction of the human interactome (See detailed analysis Supplementary Information). We have created several sets representing the human interactome: Set A) comprising only published Y2H studies from large-scale Y2H publications that contained at least 1000 interactions in the study – this interactome contains 7,722 proteins and 24,406 interactions; Set B) all Y2H data that we could identify in the public domain; this utilized 5,537 publications and includes 10,998 proteins and 47,994 interactions;

List of the Top 30 Y2H publications

pubmed:25416956 4131 13313 <-

pubmed:21988832 2521 3292 <-

pubmed:16169070 1669 3171 <-

pubmed:21900206 1106 2556 <-

pubmed:16189514 1358 2178 <-

pubmed:16713569 823 1010 <-

pubmed:14743216 259 886

pubmed:15231748 556 764

pubmed:17353931 538 760

pubmed:20211142 439 665

pubmed:23414517 453 619

pubmed:24412244 106 613

pubmed:21044950 332 610

pubmed:20936779 447 607

pubmed:25640309 192 593

pubmed:25814554 381 562

pubmed:22365833 190 557

pubmed:19549727 243 546

pubmed:23455924 345 524

pubmed:15231747 328 420

pubmed:22990118 281 339

pubmed:21163940 111 310

pubmed:21516116 359 305

pubmed:17043677 156 278

pubmed:19690564 108 268

pubmed:15604093 131 249

pubmed:18624398 182 213

pubmed:22493164 99 195

pubmed:24705354 191 191

pubmed:15383276 70 172

To increase the size of the interactome, For Set C interactome we collected the human protein-protein interaction data from the partners of the International Molecular Exchange Consortium (IMEx [4]), Phosphosite (http://www.phosphosite.org/); and structurally determined complexes from the Protein Data Bank [5] as a starting point and removed interactions that were obtained by low-confidence techniques, or that were ‘implied’ binary interactions from low-resolution experiments such as immunoprecipitation of large complexes. We compared some key parameters such as network centralization (0.04 for the large network and 0.18 for the small network) and clustering coefficient (0.065 for the large network and 0.0068 for the small network). Given that the small Y2H network remains very limited in both its representation of proteins and interactions, it is difficult with available data to judge how representative it is of the full interactome when this is finally identified.

Below is a summary of statistics of each interactome and how it affects the training sets.

For all these reasons the only possibility is to use the larger interactome to allow the statistical analysis.

To begin to address the issue of study bias, we did the following:

1. We removed isolated proteins and small isolated networks that were not connected to the main network.
2. We divided the proteins into groups depending on their number of first neighbours as described by Hase et al, 2009: low degree (≤5), medium degree (6-30) or high degree (≥31). As a result, all comparisons and predictions were carried out among proteins with comparable number of neighbours.

These approaches go some way towards balancing the data and correcting for study imbalance to some degree yet they do not eliminate it. However, the resulting, high confidence interactome contained 89,691 interactions between 13,345 proteins, and contained all 343 drug targets from our positive training set. This provides a more solid basis for statistical analysis. In future, when a more comprehensive and unbiased view of the human interactome emerges, it will better enable addressing the true effect of study imbalance.

## Features used in model construction

The data dictionary in Supplementary Table 2 in this document summarised the numbers and classes of features used for model construction. These features fall into three different categories: Topological – these contain 33 features derived from the network topology such as degree, Community-based (185 features) and Graph-based (103 features). As discussed in the main text, many of these features can be correlated. To assess the degree of correlation between the different features we have calculated pairwise Pearson correlations for all the 51,360 feature pairs. The full data are provided in the Supplementary File: ‘S2_File.xlsx’. In summary, most feature pairs show very little correlation. With the exception of highly correlated graphlets and orbits, 461 feature pairs show positive correlation (>0.6) and only 72 feature pairs show significant negative correlation (<-0.6). Supplementary Figure 6a shows a heatmap of Pearson correlation values. Green=positive correlation, red= negative correlation and black is no correlation. Interestingly, the region of greatest correlation contains the graphical features. This is because simpler graphlets are also subsets of more complex graphlets. Supplementary Figure 6b shows the distribution of correlation values highlighting that most feature pairs are not correlated.

To begin to assess which features are most associated with the drug target classes, namely all drug targets, targets of cancer drugs, or targets of other therapeutic areas, we calculate ANOVA or Fisher’s exact test p-values supplied in the supplementary file: S3_File.xlsx). We find that 218 feature-target class pairs are significantly associated (P-value < 0.05, Bonferroni corrected for false discovery). The most prevalent association occurs between graphlets and the class Cancer Drug Targets (87 out of 104 significant associations). In sharp contrast, the class Drug Targets of Other Therapeutics is completely devoid of this association, but is enriched in a number of community features (8 out of 10 associations). This uneven feature type enrichment is cumulative in the All Drug Targets class. The number of statistically significant associations between feature types and target classes is shown in the following table:

|  | All Drug Targets | Cancer Drug Targets | Drug Targets Other TAs |
| --- | --- | --- | --- |
| Topological | 15 | 14 | 2 |
| Community-based | 11 | 3 | 8 |
| Graphical | 78 | 87 | 0 |

## Validation of predictive models

Supplementary Table 3 shows the average cross-fold validation AUC results across all three algorithms for each dataset, which vary between 57-86% with a mean across datasets of 75%. The ‘Low’ subsets of each category, which include proteins with <6 first neighbors showed the worst predictive accuracy, reflective of the small size of these datasets compared to the ‘All’, ‘Medium’ and ‘High’ datasets. All predictions and scores are provided in Supplementary Table 3.

To further validate the results and overcome the caveats of 10-fold cross validation in a network (discussed in Methods section), a random class dataset was created: 343 proteins were randomly labeled as positive and the three predictive modeling algorithms were applied. The average 10 fold cross-validation results are significantly lower than the accuracy results from the real datasets (average 50%) and range between 48% for the ‘All dataset to 51% for the ‘High’ dataset providing increased confidence in the discriminatory ability of the models (Supplementary Table 3).

As no negative training set exists, we provide the prediction results as a rank against the whole interactome. As this our training is a PU (Positive-Unlabelled) training exercise (lacking a negative training set), a classical precision-recall analysis is not possible, hence we use the Area Under The Curve (AUC) as the measure for accuracy. To visualise the recall power, we have calculated the sensitivity (the number of true positives predicted) as a function of recall (all positives predicted) for each of the models sets, in comparison with random (Supplementary Figure 5.) The prediction strength of druggable targets (all therapeutic areas) against random has a p-value < 2e-16; the prediction strength of druggable targets (cancer) against random has a p-value 3.69e-14; and the prediction strength of druggable targets (non-cancer) against random has a p-value < 2e-16. Based on the response curves in Supplementary Figure 5, we have selected the 25% recall cut-off (equivalent to 75% centile rank) as the cut off for our positive predictions as it corresponds to 97% precision for cancer target predictions, 90% precision for druggable target predictions (all therapeutics areas) and 90% precision for non-cancer target predictions.

**GBM:**

Grid Search of GBM parameters over a 10-fold cross-validation. Shrinkage = 0.01

| **Neighbours** | **Target** | **Distribution** | **No Trees** | **Depth** | **Min Obs** | **Avg AUC** |
| --- | --- | --- | --- | --- | --- | --- |
| All | All Targets | bernoulli | 376 | 15 | 5 | 0.8059 |
| All | Other TAs | bernoulli | 204 | 15 | 10 | 0.7791 |
| All | Cancer Targets | bernoulli | 357 | 15 | 3 | 0.8576 |
| All | Cancer-Associated | bernoulli | 411 | 15 | 5 | 0.8175 |
| Low | All Targets | bernoulli | 49 | 15 | 10 | 0.6420 |
| Low | Other TAs | bernoulli | 44 | 15 | 10 | 0.7369 |
| Low | Cancer Targets | adaboost | 1 | 15 | 3 | NaN |
| Low | Cancer-Associated | bernoulli | 25 | 15 | 10 | 0.6413 |
| Medium | All Targets | bernoulli | 194 | 15 | 3 | 0.7507 |
| Medium | Other TAs | bernoulli | 106 | 15 | 5 | 0.7159 |
| Medium | Cancer Targets | bernoulli | 98 | 15 | 10 | 0.6862 |
| Medium | Cancer-Associated | bernoulli | 265 | 15 | 5 | 0.6914 |
| High | All Targets | bernoulli | 128 | 15 | 5 | 0.7494 |
| High | Cancer Targets | bernoulli | 130 | 15 | 5 | 0.8200 |
| High | Cancer-Associated | bernoulli | 203 | 15 | 3 | 0.7060 |
|  |  |  |  |  |  |  |

**GLMNET**

Grid Search of GLM parameters over a 10-fold cross-validation

Elasticnet Ridge (alpha=0) and Lasso (alpha =1) and mix (alpha =0.5) nlambda =1000

| **Neighbours** | **Target** | **Alpha** | **Alpha Type** | **Avg AUC** |
| --- | --- | --- | --- | --- |
| All | All Targets | 0.75 | Mostly Lasso | 0.8306 |
| All | Other TAs | 0 | Ridge | 0.7933 |
| All | Cancer Targets | 0 | Ridge | 0.8631 |
| All | Cancer-Associated | 0 | Ridge | 0.8330 |
| Low | All Targets | 0.5 | Mix | 0.5737 |
| Low | Other TAs | 0 | Ridge | 0.6771 |
| Low | Cancer Targets | 0 | Ridge | NaN |
| Low | Cancer-Associated | 0.5 | Mix | 0.6316 |
| Medium | All Targets | 0 | Ridge | 0.7812 |
| Medium | Other TAs | 0.5 | Mix | 0.7903 |
| Medium | Cancer Targets | 0.75 | Mostly Lasso | 0.7522 |
| Medium | Cancer-Associated | 0 | Ridge | 0.7323 |
| High | All Targets | 0.75 | Mostly Lasso | 0.8708 |
| High | Cancer Targets | 0 | Ridge | 0.9831 |
| High | Cancer-Associated | 0 | Ridge | 0.9321 |
|  |  |  |  |  |

**Random Forest**

| **Neighbours** | **Target** | **Trees** | **Avg AUC** |
| --- | --- | --- | --- |
| All | All Targets | 100 | 0.7918 |
| All | Other TAs | 100 | 0.7618 |
| All | Cancer Targets | 100 | 0.8483 |
| All | Cancer-Associated | 100 | 0.7913 |
| Low | All Targets | 100 | 0.7110 |
| Low | Other TAs | 100 | 0.7031 |
| Low | Cancer Targets | 100 | NaN |
| Low | Cancer-Associated | 100 | 6203 |
| Medium | All Targets | 100 | 0.6984 |
| Medium | Other TAs | 100 | 0.6839 |
| Medium | Cancer Targets | 100 | 0.7107 |
| Medium | Cancer-Associated | 100 | 0.6693 |
| High | All Targets | 100 | 0.7523 |
| High | Cancer Targets | 100 | 0.8277 |
| High | Cancer-Associated | 10 | 0.6886 |

## Predictive power of individual features derived from LASSO and EN models

The supplementary file ‘S1_File.tar.gz’ details the results and relative information content of each of the topological, community and graphical features used to train the models.

## Drug Combination Studies

To begin to explore associations between network environments and drug combinations we examined published synergistic drug combinations that have been experimentally verified [6,7] as they are more likely to be observed than for combination of drugs acting on targets with different network environments [6,8,9]. Additionally, we examined the Drug Combinations database DCDB2.0 [10] for reported combinations of targeted therapies. We found, as expected from reports of clinical data, that most entries in DCDB report combinations with cytotoxic chemotherapies which are unsuitable for this analysis. Additionally, no reports of synergy or continuation of response are reported in the database. The only report is whether a particular drug combination was found to be clinically efficacious – thus again making the data unsuitable for our analysis. Nonetheless we identified some reported efficacious combinations between kinase inhibitors. Most where combinations of polypharmacologic multi-kinase inhibitors such as sunitinib and lapatinib. However, interesting combinations included the EGFR inhibitor gefitinib and the HMGCOR inhibitor simvastatin; as well as combinations between kinase inhibitors and the aromatase inhibitor, letrozole. Further clinical or experimental investigation is required to identify whether these combinations are synergistic and whether any synergy is long lived.

| **Combination** | **Indication** | **Report** |
| --- | --- | --- |
| Gefitinib: 250 mg; Simvastatin: 40 mg | Lung Cancer | Efficacious |
| Sirolimus: 1 mg/kg/d; Imatinib: 10 mg/kg/d | Preventing restenosis after intimal injury | Efficacious |
| Sorafenib: 400 mg; Sunitinib: 50 mg | Renal Cell Carcinoma | Efficacious |
| Gefitinib: 250 mg; Sunitinib: 37.5 mg | Carcinoma, Renal Cell | Efficacious |
| Dasatinib: PKC412 = 1:200 | Mast cell leukemia (MCL) | Efficacious |
| Lapatinib; 1.5 g; Letrozole: 2.5 mg | Postmenopausal Hormone Receptor-Positive Metastatic Breast Cancer | Efficacious |
| Gefitinib: 0.1 mcM/L; ST1926: 0.01 mcM/L | Neuroblastoma | Efficacious |
| Lapatinib: 1.5 g/d; Letrozole: 2.5 mg/d | Neoplasms, Breast | Efficacious |
| Bevacizumab: 10 mg/kg; Lapatinib: 1.5 g | Neoplasms, Breast | Efficacious |

## References

1. Przulj N (2007) Biological network comparison using graphlet degree distribution. Bioinformatics 23: e177–e183. doi:10.1093/bioinformatics/btl301.

2. Rual J-F, Venkatesan K, Hao T, Hirozane-Kishikawa T, Dricot A, et al. (2005) Towards a proteome-scale map of the human protein-protein interaction network. Nature 437: 1173–1178. doi:10.1038/nature04209.

3. Rolland T, Taşan M, Charloteaux B, Pevzner SJ, Zhong Q, et al. (2014) A proteome-scale map of the human interactome network. Cell 159: 1212–1226. doi:10.1016/j.cell.2014.10.050.

4. Orchard S, Orchard S, Kerrien S, Kerrien S, Abbani S, et al. (2012) Protein interaction data curation: the International Molecular Exchange (IMEx) consortium. Nat Methods 9: 345–350. doi:10.1038/nmeth.1931.

5. Gutmanas A, Alhroub Y, Battle GM, Berrisford JM, Bochet E, et al. (2014) PDBe: Protein Data Bank in Europe. Nucleic Acids Res 42: D285–D291. doi:10.1093/nar/gkt1180.

6. Held MA, Langdon CG, Platt JT, Graham-Steed T, Liu Z, et al. (2013) Genotype-selective combination therapies for melanoma identified by high-throughput drug screening. Cancer Discov 3: 52–67. doi:10.1158/2159-8290.CD-12-0408.

7. Gowrishankar K, Snoyman S, Pupo GM, Becker TM, Kefford RF, et al. (2012) Acquired resistance to BRAF inhibition can confer cross-resistance to combined BRAF/MEK inhibition. J Invest Dermatol 132: 1850–1859. doi:10.1038/jid.2012.63.

8. Al-Lazikani B, Workman P (2013) Unpicking the combination lock for mutant BRAF and RAS melanomas. Cancer Discov 3: 14–19. doi:10.1158/2159-8290.CD-12-0520.

9. Clendening JW, Penn LZ (2012) Targeting tumor cell metabolism with statins. Oncogene 31: 4967–4978. doi:10.1038/onc.2012.6.

10. Liu Y, Wei Q, Yu G, Gai W, Li Y, et al. (2014) DCDB 2.0: a major update of the drug combination database. Database (Oxford) 2014: bau124. doi:10.1093/database/bau124.
